# Sugar Alcohols and Organic Acids Synthesis in *Yarrowia lipolytica*: Where Are We?

**DOI:** 10.3390/microorganisms8040574

**Published:** 2020-04-15

**Authors:** Patrick Fickers, Hairong Cheng, Carol Sze Ki Lin

**Affiliations:** 1 Microbial Process and Interactions, TERRA Teaching and Research Centre, University of Liege—Gembloux Agro-Bio Tech, 5030 Gembloux, Belgium; 2 State Key Laboratory of Microbial Metabolism, School of Life Sciences & Biotechnology, Shanghai Jiao Tong University, Shanghai 200240, China; chrqrq@sjtu.edu.cn; 3 School of Energy and Environment, City University of Hong Kong, Tat Chee Avenue, Kowloon, Hong Kong; carollin@cityu.edu.hk

**Keywords:** *Yarrowia lipolytica*, organic acids, polyols, sugar alcohols

## Abstract

Sugar alcohols and organic acids that derive from the metabolism of certain microorganisms have a panoply of applications in agro-food, chemical and pharmaceutical industries. The main challenge in their production is to reach a productivity threshold that allow the process to be profitable. This relies on the construction of efficient cell factories by metabolic engineering and on the development of low-cost production processes by using industrial wastes or cheap and widely available raw materials as feedstock. The non-conventional yeast *Yarrowia lipolytica* has emerged recently as a potential producer of such metabolites owing its low nutritive requirements, its ability to grow at high cell densities in a bioreactor and ease of genome edition. This review will focus on current knowledge on the synthesis of the most important sugar alcohols and organic acids in *Y. lipolytica*.

## 1. Introduction

*Yarrowia lipolytica* is an ascomycetous yeast generally recognized as safe (GRAS) status [1,2]. Due to its ability to catabolize hydrophobic substrates (i.e., alkanes, triglycerides and fatty acids) for the production of single-cell proteins, interest in this yeast began in early 1970 [3]. *Y. lipolytica* is also known for its ability to produce and secrete enzymes naturally such as the lipase lip2p, proteases and RNases at high quantities [1,4], but also a panoply of metabolites such as organic acids and sugar alcohols. The release of the 20 Mb of its genome in 2004, and subsequent development of efficient genome editing tools have enabled the development of metabolic engineering strategies for the production of recombinant proteins and metabolites of biotechnological interest [5,6,7]. These engineering strategies also aimed to endow *Y. lipolytica* with features for the catabolism of complex carbohydrates contained in organic wastes generated from industries or agricultural practices [8]. In this review, we aim to summarize the main research that has been performed, both at the molecular (strain development) and production (bioreactor) levels, for the synthesis of the most important organic acids and sugar alcohols using *Y. lipolytica*.

## 2. Organic Acids

**Figure 1:**
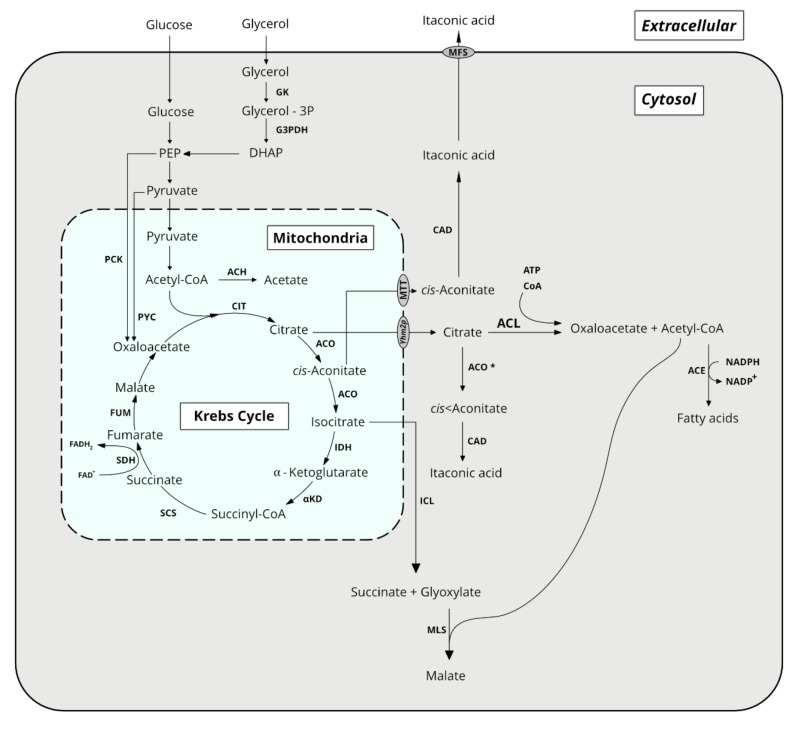
Overview of the principal metabolic pathways for organic acid synthesis in *Y. lipolytica*. ACL: ATP citrate lyase; ACC: acetyl CoA carboxylase; ACO: aconitase; ACO *: truncated aconitase (for cytosol localization); ACH: acetyl CoA hydrolase; CAD: cis-aconitate decarboxylase; CIT: citrate synthase; DHAP: dihydroxyacetone-P; FUM: fumarase; GK: glycerol kinase; G3PDH: glycerol-3P dehydrogenase; ICL: isocitrate lyase; IDH: isocitrate dehydrogenase; MLS: malate synthase; PEP: phosphoenol pyruvate; PYC: pyruvate carboxylase; SCS: succinyl CoA synthetase; SDH: succinate dehydrogenase; PCK: phosphoenolpyruvate carboxykinase; αKD: αketoglutarate dehydrogenase.

All organics acids discussed herein derive directly or indirectly from the Krebs cycle (Figure 1). Their synthesis is thus directly linked to energy formation, rendering metabolic engineering strategies to enhance their production more complex. It is also interconnected to synthetic pathways of lipids and other important molecules. Therefore, culture strategies aim to direct the carbon flux through the synthesis of these organic acids while trying to minimize the loss of energy and building elements in those side pathways. The main organic acids produced by *Y. lipolytica* are presented in Section 2.1, Section 2.2, Section 2.3, Section 2.4 and Section 2.5.

### 2.1. Citric Acid

Citric acid (CA) is a weak organic acid present in citrus fruits. It is also an intermediate of the Krebs cycle that occurs in all-aerobic microorganisms. Its worldwide production in 2020 is estimated to two million tonnes with China as the main producer [9]. About 70% of the total CA production is used by the food industry, 20% in detergent and the remaining 10% by the chemical and pharmaceutical industries [10]. As a food additive, CA is mainly used as an antioxidant, flavoring agent, acidifier and preservative. It is produced mainly by fermentation processes from industrial by-products in order to reduce the production cost. Historically, *Aspergillus niger* has been used for industrial CA production [9]. Yeasts have been also reported as CA producers, and among them, *Y. lipolytica* has been described as one of the most promising species [10]. The main drawback of using *Y. lipolytica* for CA production is its propensity to produce high amount of iso-CA (iCA) [11]. One of the key parameters for CA accumulation in yeasts is linked to the deficiency of nitrogen in the culture broth, as citrate synthase is negatively regulated by ammonium [12]. In *Y. lipolytica*, nitrogen starvation induces the activity of an adenosine monophosphate deaminase that cleaves AMP, a necessary cofactor for isocitrate dehydrogenase (IDH). This results in a disrupted Krebs cycle with accumulation of early metabolites in the mitochondria, such as citric acid [13,14]. Citric acid can be finally exported from the mitochondria by exchange of cytosolic oxaloacetate via the specific transporter Yhm2p to accumulate extra-cellularly [15]. Therefore, at high C/N ratio, the excess of carbon is redirected toward CA synthesis during the stationary phase. This has been demonstrated experimentally by several authors. For the *Y. lipolytica* stain NRRL YB-423, the optimal C/N ratio for high CA production rate is 172, while a ratio of 343 is optimal to maximize both rate and yield [16].

Being an oleaginous yeast, *Y. lipolytica* is able to accumulate large quantities (over 50%) of intracellular lipids mainly as triacylglycerol [1]. In these yeasts, de novo lipid synthesis and accumulation are triggered by C/N imbalance as it has been demonstrated that CA is the precursor for lipid synthesis [17]. Citrate is cleaved by the ATP-citrate lyase, an enzyme specific to oleaginous yeast, into acetyl coenzyme A and oxaloacetate. Acetyl-CoA is the substrate of acetyl-CoA carboxylase involved in fatty acid synthesis. Therefore, both nitrogen starvation and excess carbon could lead to CA production or lipid accumulation. Ochoa-Estopier and Guillouet (2014) demonstrated a C/N ratio of 11.7 yields to lipid accumulation while a ratio of 47.6 favors CA production using D-stat continuous cultivation techniques (D-stat) [17]. In *Y. lipolytica*, CA productivity relies also on the nature of the carbon, nitrogen and phosphate sources used as nutrients but also on the culture conditions (pH, oxygen transfer, temperature). Several authors pointed out the importance of the pH of the culture medium for efficient CA production [18,19,20]. Zhang et al. (2019) proposed some hypotheses that support those observations [21]. In *Y. lipolytica*, pH differentially affects lipid and CA synthesis. Lipid synthesis is favored at an acidic condition, while CA production is more triggered at neutral pH. This has been linked with CA transport since transporter genes are upregulated at neutral pH. Moreover, thermodynamic calculations suggested that CA secretion is more energetically favorable, assuming the CA fully protonated is the form for secretion [21]. Dissolved oxygen (DO) concentration (more precisely oxygen transfer rate) is also an important parameter to consider for CA production. Ferreira et al. (2016) demonstrated that at a higher oxygen volumetric mass transfer coefficient (K_l_a), CA titer was increased significantly (almost 8-fold) for the *Y. lipolytica* strain W29 grown on glycerol [22]. For *Y. lipolytica* strains Wratislavia 1.31 and Wratislavia AWG7, the highest CA yields were reported at DO of 40% of saturation [23]. Recently, it has been demonstrated that DO impact on CA titer depends on the carbon source used [24]. The control of DO at 50% of saturation significantly enhances CA production on glucose and glucose/glycerol media, while it has no effect on a pure glycerol-based medium. The influence of the growth rate on CA production was investigated in chemostat cultures. An increase of the dilution rate, and thus the growth rate from 0.009 to 0.031 h^−1^, led to a decrease in CA titer from 86.5 to 51.2 g/L [25]. In contrast, productivity and yield increased from 0.78 to 1.59 g/(L·h) and from 0.59 and 0.61 g/g, respectively.

Production of CA strongly relies on the strain selection. This has been investigated by several authors [16,26]. More recently, Carsanba et al. (2019) screened a collection of wild-type strains for CA production [10]. The productivities obtained ranged from 0.002 to 0.029 g/(g·h), corresponding to a final CA concentration in the culture supernatant of 0.48 and 20.47 g/L, respectively. That author also tested different C/N ratios (167, 367, 567), using glucose as the carbon source. In a bioreactor, the highest CA titer and yield obtained were at C/N of 367 (i.e., 72 g/L and 0.77 g/g, respectively). In contrast, the highest CA productivity was obtained at a C/N ratio of 567 (i.e., 0.06 g/(g·h)).

CA production from different industrial waste substrates has been investigated for both wild-type or engineered strains. Papanikolaou et al. (2002) reported that the *Y. lipolytica* strain LGAMS(7)1 was able to produce in shake-flasks CA up to 35 g/L and a resultant yield of 0.44 g/g, starting from 120 g/L of carbon and at a C/N ratio of 388 using raw glycerol (i.e., the main waste of biodiesel production) as the carbon source [18]. When unbuffered media was used, lower CA titer and yield were obtained, thus confirming pH modulates CA productivity. In those conditions, lipid accumulation was less than 10% (*w*/*w*). With the *Y. lipolytica* strain, VKM Y-2373 grown in a bioreactor with feeding of glycerol-containing wastes from biodiesel industry, CA titer and yield were of 67.7 g/L and 0.59 g/g, respectively [27]. In those conditions, CA/iCA ratio was 1.7:1. Using a repeated batch strategy (cell recycling), CA titer of 107 g/L on average was obtained with a productivity and yield of 1.42 g/(L·h) and 0.64 g/g, respectively [28]. In this cultivation strategy, 30% of the medium was replaced every 3 days. The culture system remained stable over 42 days. Similar experiments performed with the acetate negative mutant strain Wratislavia AWG7, an average CA titer of 157 g/L was obtained, with a productivity and yield of 1.05 g/(L·h) and 0.78 g/g, respectively [28]. In those conditions, the system remained stable for over 16 cycles, corresponding to a total culture time of 1650 h. Mixtures of glucose and glycerol as carbon substrate have been also tested for CA production. For a C/N ratio in the range of 90–98, a lower CA titer (9.1 g/L) was obtained when a mixture of glucose and glycerol was used as compared to pure solutions of glucose or glycerol (11.9 and 13.7 g/L, respectively). By contrast, a higher yield was obtained for the carbon mixture, i.e., 0.75 g/g versus 0.66 and 0.64 g/g, respectively [18]. The CA titer and lipid accumulation were compared for a mixture of glucose and free-fatty acid (stearin) versus glucose only in fermentations using strain ACA_DC50109 [29]. A lower lipid accumulation at a low stearin concentration was obtained. Likewise, CA productivity and yield were remarkably higher in absence of fatty acids as a co-substrate to glucose. Using the Plackett–Burman design, the production of CA in solid-state fermentation (SSF) by *Y. lipolytica* NCIM3589 from pineapple waste have been optimized [30]. In the optimal experimental conditions, 202 g CA was produced per kg of dry pineapple wastes. This result demonstrated that significant CA titer could be obtained from agronomic wastes by the SSF process. The olive processing industry generates a liquid fraction still rich in lipids, known as olive-mill wastewater (OMW). In OMW (30% *v*/*v*) blended with 65 g/L glucose, CA titer of 28.9 g/L and a yield of 0.53 g/g glucose was obtained for the *Y. lipolytica* strain ACA-DC 50109 [31]. This value is higher than those obtained in a glucose-based medium, suggested that OMW triggers CA synthesis and this waste stream could be used as a co-substrate for CA production. Using a mixture of OMW and crude glycerol, the strain ACA-DC 5029 produced CA with a resultant titer of 79 g/L and yield from glycerol of 0.46 g/g in 528 h [32]. For the *Y. lipolytica* strain ACA-YC 5031, addition of NaCl (%) in OMW-based media significantly increases CA production with titer and yield from glycerol of 54 g/L and 0.82 g/g, respectively [33]. Using 100 g/L sunflower oil as a carbon source, CA titer of 66.2 and 50 g/L were obtained for the *Y. lipolytica* strain TEM YL 3 and TEM YL 20, respectively [34]. Glucose hydrola is a byproduct of glucose manufacturing from potato starch, and it was used for CA production by different strains of *Y. lipolytica* [27]. In those conditions, the highest CA titer (100 g/L) was attained in 80 h, with a productivity of 1.25 g/(L·h) and yield from glucose of 0.93 g/g. Inulin is a fructose polymer found in different plants, it has been used as a carbon source for CA production. As *Y. lipolytica* cannot metabolize inulin naturally, heterologous expression of the *INU1* gene from *Kluyveromyces marxianus* has been considered to obtain a recombinant strain with inulinase activity at the cell surface. With such a strategy, Liu et al. (2010) obtained a CA titer of 68.9 g/L after 313 h of cultivation in a 2-L bioreactor containing 100 g/L pure inulin [35]. When the strain was grown in a 10-L bioreactor in the presence of extract of Jerusalem artichoke tubers (i.e., with a total sugar concentration of 84 g/L), CA titer reached 68.3 g/L with a yield of 0.91 g/g within 336 h [36]. With the strain *Y. lipolytica* AWG7 INU 8, overexpressing *INU1* gene from *K. marxianus* CBS6432, a CA titer of 203 g/L was obtained by repeated batch culture; this corresponded to a productivity and yield of 0.51 g/(L·h) and 0.85 g/g, respectively [37]. In addition, sucrose was tested as a carbon source for CA production with *Y. lipolytica* strain A101-B56-5 overexpressing invertase (*SUC2* gene from *Saccharomyces cerevisiae*). CA titer and yield based on sucrose as a carbon source were 45 g/L and 0.64 g/g, respectively in a 5-L bioreactor after 72 h. In those conditions, iCA synthesis was below 2% [38]. By overexpressing xylose reductase and xylitol dehydrogenase from *Scheffersomyces stipites* together with endogenous xylulokinase, CA titer of 80 g/L could be obtained from a xylose-based medium [39]. However, the CA yield based on xylose as a carbon source was only 0.24 g/g.

In addition, endogenous cell metabolism has been optimized for a carbon source uptake in order to enhance CA production. Glycerol is one of the preferred carbon sources of *Y. lipolytica* [40]. Upon overexpression of glycerol kinase (GK, *YALI0F00484g*) and glycerol-3-P dehydrogenase (GDH, *YALI0B02948g*), glycerol uptake rate was increased by 25% in the *Y. lipolytica* strain A101 [41]. In a 5-L bioreactor and using pure glycerol, this allowed a 14-fold increase in CA productivity and yield (i.e., 0.69 g/(L·h), 0.43 g/g, respectively) as compared to the wild-type strain A101. Using the same strain but cheap raw glycerol as a main carbon source, CA titer of 34.1 g/L was obtained; corresponding to a productivity and yield of 0.23 g/(L·h) and 0.22 g/g, respectively [42].

Different authors reported specific genetic modifications with the aim to increase CA production. Overexpression in multi-copies of *ICL1* (*YALI0C16885g*) encoding isocitrate lyase resulted to a strong shift of the CA/iCA ratio in favour of CA [43]. On carbon sources such as sucrose, glycerol and glucose, the iCA proportion decreased from 10–12% to 3–6%, while it decreased from 37–45% to 4–7% on sunflower oil or hexadecane without influencing the total amount of acids produced (CA and iCA). Using a *Y. lipolytica* SWJ-1b strain disrupted for gene *ACL1* (*YALI0E34793g*) encoding ATP-citrate lyase (ACL) and overexpressing endogenous *ICL1* and *K. marxianus INU1* gene, a CA titer of 84 g/L (1.8 g/L iCA) was obtained from inulin (10%). In those conditions, the CA yield from inulin was 89.6% [44]. In the same strain, disruption of *ACL1* and overexpression of *ICL1*, reduced iCA and lipid synthesis, and promoted CA production from inulin [44]. During cultivation in a 2-L bioreactor in the presence of 10% inulin, CA titer of 84 g/L was obtained within 214 h with a yield of 0.89 g/g. Pyruvate carboxylase (*YALI0C24101g*, *PYC*) catalyzes the carboxylation of pyruvate into oxaloacetate in the cytosol in the presence of CaCO_3_ used as a source of carbon dioxide [45]. Expression of pyruvate carboxylase from *Meyerozyma guiliermondii* in *Y. lipolytica* SWJ-1b resulted in an increased CA titer of 101 g/L (with a resultant yield from glucose 0.89 g/g) within 240 h. Co-expression of *YHM2* (*YALI0B10736g*) and *AMDP* encoding, respectively, mitochondrial citrate carrier and adenosine monophosphate deaminase in strain W29 allowed a CA titer and yield from glucose of 97.1 g/L and 0.5 g/g during culture in bioreactor [15]. In those conditions, the CA productivity was 0.93 g/(L·h). Disruption of 2-methyl citrate dehydratase (*YALY0F02497g, PHD1*) that converts 2-methylcitrate into 2methyl-cis-aconitate in the 2-methylcitrate cycle of propionate metabolism was used to mimic nitrogen starvation [46]. The resulting strain JMY1203 (*Δphd1*) produced CA with titer and yield of 26 g/L and 0.57 g/g, respectively, from glycerol under nitrogen excess conditions.

CA has been also produced in non-aseptic operational conditions. With alginate immobilized cold adapted and lactose positive *Y. lipolytica* strain B9, CA was produced from partly deproteinized cheese whey after temperature treatment for 15 min at 90 °C. In optimal operational conditions (20 °C, pH 5.5, lactose content 20 g/L), CA was produced with titer of 33 g/L after 120 h of culture. In those conditions, the CA/iCA ratio was equal to 6.8 [47]. Non-aseptic OMW enriched with glycerol (50 g/L) was also used as substrates for CA production by the *Y. lipolytica* strain LGAM s(7) in the bioreactor (5 L working volume) at 25 °C and pH controlled at 2.8. In those condition, CA was produced after 300 h of culture with titer and yield of 21 g/L and 0.68 g/g, respectively [48]. The process of CA synthesis using the *Y. lipolytica* strain ACA-YC5033 in aseptic and non-aseptic conditions were compared in terms of productivity and yield using OMW and glucose mixtures as substrates [49]. Equivalent CA titer and yield, i.e., 52 g/L and 0.64 g/g, respectively were obtained in both experimental conditions from a glucose concentration of 80 g/L. From all these strategies, the highest CA titer reported was around 200 g/L, but most of the CA titers were below 100 g/L (Table 1).

### 2.2. Iso-Citric Acid

Isocitric acid (iCA) is a structural isomer of CA with the difference in the hydroxyl group position. Having two asymmetric carbons, iCA has four stereoisomers with 2R,3S-iCA being an intermediate of the Krebs cycle. It is produced from cis-aconitate by the action of aconitase (i.e., aconitate hydratase as shown in Figure 1) in the presence of iron ions [50]. iCA is also consumed as a substrate by isocitrate dehydrogenase to generate α-ketoglutarate (α-KG) and by isocitrate lyase, to generate glyoxylate and succinate. Isocitrate dehydrogenase is the least active enzyme of the Krebs cycle, and it represents the bottle neck for further conversion of iCA into α-KG. Therefore, this could explain CA and iCA accumulation, especially in excess of a carbon source and starvation in nitrogen (i.e., high C/N ratio). iCA oversynthesis is related with high catalytic activities of aconitase and citrate synthase and low activities of isocitrate lyase and isocitrate dehydrogenase (Figure 1) [50]. It is also related to the continuous replenishment of oxaloacetate from pyruvate by pyruvate carboxylase (PYC, Figure 1) and through the glyoxylate pathway when cells are grown on fatty acid, ethanol or *n*-alkane [50].

Although less developed than those based on CA, applications for iCA has emerged more than 15 years ago. Finogenova et al. (2005) reported firstly the efficiency of iCA in the resorbing of blood clots and in the treatment of iron-deficiency anemia [51]. Very recently, its derivative trimethyl-iCA was proposed as a new drug for the treatment of Parkinson’s disease associated to the DJ-1 gene dysfunction [52]. iCA is also used as a synthon in the chemical and pharmaceutical industries [53]. For instance, potassium isocitrate is used for the synthesis of HIV protease inhibitors Darunavir and Brecanavir [54]. In rats, the ability of iCA to mitigate the neurotoxic effects of molybdenum and lead has been demonstrated [50].

In the *Y. lipolytica* wild-type strain, iCA is generally co-produced with CA in a proportion that is highly dependent on the strain considered, the nature of C and N substrates and their proportion. When cells are grown on carbohydrate or glycerol, iCA could represent up to 16% of the CA produced, while this proportion could represent nearly 50% on gluconeogenic substrates [55]. It also depends on the cultivation conditions (pH, dissolved oxygen) as demonstrated for CA production from ethanol, where iCA proportions ranging from 35 to 67% were found [56]. Production of iCA depends also on the DO level in the culture medium, and this dependence was found related to the carbon source considered. On a glycerol-based medium, the maximal iCA productivity was obtained at a DO of 70% of saturation [42]. In an ethanol-based medium, a DO of 95% yields to equal production of iCA and CA, while DO values between 60–65% promote the overproduction of iCA over CA [56]. In the optimized process of iCA production, DO value is modulated according the carbon source and cultivation phase considered [19]. It ranges between 20–25% during the growth phase and is increased to 50–55% during the iCA production phase. In those conditions, 90 g/L iCA was produced in 144 h with a yield from ethanol of 0.77 g/g. The production of iCA from ethanol and rapeseed oil by the *Y. lipolytica* strain VKM Y-2373 was found maximal at pH 6 [19,57]. In contrast, CA production was maximal at neutral pH [21]. Carbon sources considered for iCA synthesis were n-alkane [58], rapeseed oil [59], ethanol [19], sunflower oil [60], frying oil waste [61], straw hydrolysate [61], milk whey [47] or raw glycerol [18,42].

As stated above, aconitase converts CA to iCA via cis-aconitate intermediate. Addition of Fe^2+^ salts in the culture medium (i.e., up to 6 mg/L) led to enhancement of both iCA and CA excretion, and shifted the iCA/CA balance in favor of iCA [19,57]. This shift could be also triggered by addition of analogues of the iCA lyase reaction product in the culture medium, namely oxalic and itaconic acid. This was evidenced by Kamzolova et al. (2016) for the strain VKM Y-2373 grown on ethanol as a main carbon source. In the control culture, they obtained a CA and iCA titer of 62 and 18.3 g/L, respectively, whereas in the presence of 40 mM itaconic acid, the corresponding titers were equal to 82.7 and 28 g/L, respectively [62]. A similar effect was observed for oxalic acid, however in a lesser extent (70.0 and 23 g/L, respectively). With the *Y. lipolytica* wild-type strain 704, an iCA titer of 66 g/L was obtained with a ratio iCA/CA of 4:1 [56], while with repeated batch cultures of the strain VKM Y-2373 in a 10-L bioreactor, iCA titer of 109.6 g/L was obtained with a yield of 0.8 g/g ethanol and productivity of 1.35 g/(L·h) [54]. During trials in a 150-L bioreactor using the *Y. lipolytica* strain 704 grown in media based on paraffins and ammonium sulfate as carbon and nitrogen sources, an iCA titer of 84 g/L was obtained with a yield of 1.2 g/g after 3 days of culture [58].

Furthermore, strain improvement has been considered as one of the feasible strategies to increase iCA productivity. It was started with classical UV and/or chemical mutagenesis. From the strain VKM Y-2373, a mutant obtained by UV and NTG mutagenesis (designated mutant UV/NTG), grown in a 500 L bioreactor, an iCA titer of 88.7 g/L (i.e., 85% of total organic acid) was obtained after 6 days with a yield from rapeseed oil of 0.9 g/g [59]. In addition, genetic engineering has been employed to obtain iCA producers. Overexpression of *ACO1* (*YALI0D09361g*) gene encoding aconitase in *Y. lipolytica* H222 led to a drastic shift of the iCA/CA ratio toward iCA when sunflower oil was used as the main carbon source. The iCA content ranged from 35% to 49% for the wild-type strain to 71% for an *ACO1* multicopy transformant without any modification of the total citric acid isomer production [55]. In contrast, the shift from CA to iCA was moderate (from 10 to 17%) on sucrose, glucose or glycerol-based media. With the same strain cultivated in a bioreactor on sunflower oil, an iCA titer of 68.4 g/L, corresponding to more than 75% of total acids produced was reported [60]. This corresponded to a yield of 0.64 g/g and a productivity of 0.47 g/(L·h). Overexpression of gene *ACO1* in the *Y. lipolytica* strain 672 yielded to an iCA proportion of 75.6% and a titer of 72.6 g/L [63]. In the strain H222, disruption of *ICL1* gene encoding isocitrate lyase resulted in a slight shift (i.e., from 2% to 5%) toward iCA production when cells are cultivated on glucose and glycerol-based media [43]. Compared to CA, iCA titer was lower with a maximum value of 109 g/L was reported. However, in most cases, the iCA titer was in the range of 60–80 g/L.

### 2.3. Succinic Acid

Succinate is an intermediate of the Krebs cycle, synthetized from succinyl-CoA by succinyl-CoA synthetase (SCS) and further transformed by succinate dehydrogenase (SDH) into fumarate (Figure 1). The SDH complex consists of five subunits, namely flavoprotein (*SDH1, YALI0D11374g*), ironsulfur (*SDH2, YALI0D23397g*), cytochrome b560 (*SDH3, YALI0E29667g*), membrane anchor (*SDH4, YALI0A14784g*) and succinate dehydrogenase assembly factor 2 (*SDH5, YALI0F11957g*) [64,65].

According to the US Department of Energy, succinic acid (SA) is one of the top five platform chemicals [66]. The global market for SA in 2018 reached USD 131.7 million, and is foreseen to increase to USD 182.8 in 2023 at a CAGR of 6.8% (Markets and Markets, 2020). In 2020, SA production is estimated higher than 700 kilotons [67]. SA is currently used as ion chelator, surfactant and additive in agro-food and pharmaceutical industries. It has also applications in chemistry for the synthesis of different precursors such as adipic acid (production of Nylon X6), 1,4 butanediol (synthesis of polyesters), gamma-butyrolactone (production of pesticides and herbicides) and other green solvents and chemicals [66].

In order to improve SA productivity in *Y. lipolytica*, different authors reported the construction of mutant strains with the aim to abolish or to reduce the SDH activity and thus to lower the conversion of succinate into fumarate in the Krebs cycle. The first attempt concerned the genes *SDH1* and *SDH2* that were point mutated and disrupted, respectively [65]. These authors reported that the defect of each SDH subunit impaired cell growth on glucose but not on glycerol. Indeed, during growth of the deletion mutants on a glucose-based medium, the recycling of FAD into FADH_2_ consumed in the oxidative phosphorylation is impaired since SA was oxidized into fumaric acid. This results in less ATP synthesis in oxidative phosphorylation. In addition, the export of SA is an energy consuming process, thus increasing the ATP deficiency in the SDH mutants. In contrast, glycerol metabolism could regenerate FADH_2_ during the conversion of glycerol-3P into dihydroxyacetone phosphate by mitochondrial membrane bound glycerol-3-phosphate dehydrogenase. On a glycerol-based medium and in the presence of CaCO_3_, the SDH mutant strain was able to produce SA with a titer of 17 g/L. Subsequent breeding steps to increase cell viability yielded to a mutant Y-3314 that led to a resultant SA titer of 45.5 g/L and a yield of 0.32 g/g glycerol [68]. However, the mutant Y-3314 was still unable to grow on glucose. The same authors used a combinational approach of NTG mutagenesis and metabolic evolution to isolate the *Y. lipolytica* mutant strain Y-4215 that catabolized glucose efficiently and produced SA with a titer of 50.2 g/L in 54 h and a yield of 0.43 g/g glucose in a 3-L fed-batch bioreactor [69]. The metabolic evolution step was performed in chemostat culture during 840 h at different dilution rates (from 0.02 to 0.035 h^−1^) and pH (from 4 to 3.8). Based on a ^13^C flux balance analysis, they highlighted that 35% of the consumed glucose was directed toward the pentose phosphate pathway, and more than 84% SA was produced by the oxidative branch of the Krebs cycle. The patented strain *Y. lipolytica* VKPM Y-3753 obtained by multistep of chemical mutagenesis using NTG and subsequent two-stage selection in bioreactor resulted in a SA titer of 55.3 g/L in 48 h on a mineral glucose-based medium without pH control [70]. In addition, the reduction of the SDH activity was achieved by replacing the native promoter of *SDH2* by a regulated one, namely p*ICL1* [71]. For the resulting strain *Y. lipolytica* H222-AZ1, an increased SA production was observed, especially under oxygen limitation (i.e., 9.2 g/L). By using a weaker promoter (pPOT1), strain H222-AZ2 produced 25 g/L SA after 165 h with a productivity of 0.152 g/(L·h) and a yield from glycerol of 0.26 g/g [71]. Another engineering strategy consisted in the disruption of gene *SDH5* in the *Y. lipolytica* strain Po1f [72]. Using a fed-batch strategy based with crude glycerol, the resulting strain PGC01003 produced up to 160 g/L SA within 400 h. In situ fibrous bed bioreactor (*is*FBB) with cotton towel as cell immobilization support and a repeated batch strategy, an engineered strain PGC01003 produced 198 g/L SA with a productivity of 1.46 g/(L·h) yield of 0.42 g/g glycerol [73]. Similar to the *Y. lipolytica* engineered strain Y-3314, strain PGC01003 has an impaired glucose metabolism. The strain underwent an adaptive evolution strategy at pH 6 with the aim to restore glucose metabolism [74]. After 21 days of evolution, growth ability on glucose was significantly increased, with a specific growth rate of 0.72 h^−1^ for the evolved strain PSA02004. In 2-L batch fermentation on an YPD medium or medium based on food waste hydrolysate, SA titers of 65.7 and 87.9 g/L, respectively, were obtained within 96 h. This corresponds to productivity and yield of 0.69 g/(L·h) and 0.5 g/g on YPD and of 0.7 g/(L·h) and 0.56 g/g on food wastes.

Alkali used for pH regulation during cell growth and medium acidification for SA recovery generated large amounts of by-products such as gypsum. Moreover, it represents also the largest cost in SA production and the recovery process [75]. This can be avoided or at least reduced by performing SA production at a low pH. To solve this issue, the strain PSA02004 was further evolved to select the mutant which would be able to grow and produce SA at a low pH [76]. Repeated batch cultures in *is*FBB with decreasing pH steps from 6 to 3 (between 4 to 8 repeated batches per pH step). For the evolved strain PSA3.0, SA titer, productivity and yield from glucose of 19.3 g/L, 0.52 g/(L·h) and 0.29 g/g, respectively, were obtained at pH 3. Although these values were lower than those of the parental strain grown at pH 6, titer and yield were 4.8 and 4.6-fold higher than that achieved by strain PSA02004 at pH 3. Strain characterization demonstrated that in the evolved strain PSA3.0, the pathway from pyruvate to acetate was partly blocked as no acetate could be detected as compared to the parental strain (acetate titer of 15.7 g/L). In fed-batch culture, stain PSA3.0 produced 78.8 and 18.9 g/L SA using glucose and mixed food waste hydrolysate as the carbon source, respectively. In addition, it was demonstrated that the acetic acid overflow in the SDH negative strain was caused by a CoA-transfer reaction from acetyl-CoA to succinate in the mitochondria rather than a pyruvate decarboxylation reaction. As a validation of this hypothesis, disruption of the *ACH1* gene (*YALI0E30965g*) encoding acetyl CoA-transferase (ACH) abolished the formation of acetic acid [77]. The authors also investigated the effect of overexpressing genes encoding key enzymes of oxidative steps of the Krebs cycle, reductive carboxylation and glyoxylate bypass on SA productivity and by-products formation. For a strain overexpressing genes of phosphoenolpyruvate carboxykinase (PCK) from *Saccharomyces cerevisiae* and endogenous succinyl-CoA synthetase beta-subunit 2, the SA titer was increased by a 4.3-fold. With the resulting strain PGC202, SA titer and yield from glycerol of 110.7 g/L and 0.53 g/g were obtained in fed-batch fermentation without pH control after 138 h of cultivation [77]. However, strain PGC202 produced a large number of by-products such as mannitol (19.9 g/L). The reduction of SDH activity was also achieved by truncating the promoter of the *SDH1* gene. This strategy yielded in a drastic reduction of the Sdh activity (over 77%) without impairing glucose metabolism. The authors also further optimized the metabolic flux toward SA by overexpression of endogenous genes of the glyoxylate pathway and the oxidative branch of the Krebs cycle together with the *PCK* gene from *Actinobacillus succinogenes*. In the last step, the engineered strain was evolved to tolerate high glucose concentration. In a fed batch bioreactor at pH 5, the optimized strain ST8578 produced SA with a titer of 33.5 g/L, a yield of 0.26 g/g glucose and a volumetric productivity of 0.6 g/(L·h). For that strain, the production of mannitol as a by-product could be avoided by maintaining a neutral pH.

Different processes have been also developed to produce SA from raw materials or industrial by-products. When ethanol is used as the main carbon source, the *Y. lipolytica* wild-type strain VKM Y-2412 is able to produce indirectly SA. α-ketoglutarate (α-KG) was first produced before being decarboxylated by H_2_0_2_ gradually added in the bioreactor. After 8 days of growth, the culture broth contained SA 63.4 g/L, α-KG 0.25 g/L, acetic acid 2.9 g/L, iCA 1.7 g/L and 0.5 g/L pyruvic acid. The SA yield from ethanol was 0.58 g/g [78]. The authors used a similar strategy with the same strain to produce SA from rapeseed oil [79]. After the decarboxylation step, SA titer of 69 g/L was obtained, with coproduction of 1.36 g/L acetic acid. Okara is a by-product of soymilk and tofu manufacturing that contains 40–60% carbohydrates, 20–30% proteins and 10–20% lipids [80]. It has been used to facilitate the cell growth of *Y. lipolytica* NCYC2904 and SA production, after 5 days was 33.7 g/kg dry matter. Agricultural residues, namely corn stalk, wheat straw and sugarcane bagasse for production of SA in *is*FBB. Sugarcane bagasse was found to be the optimal cell immobilization matrix. In batch culture, SA titer was 53.6 g/L, productivity 1.45 g/(L·h) and yield 0.45 g/g in fed-batch mode, SA titer reach 209.7 g/L in a cultivation medium with an initial glycerol of 120 g/L [81].

### 2.4. α-Ketoglutaric Acid

α-ketoglutaric (α-KG) is an intermediate of the Krebs cycle, which is used in food, medicine, chemical and cosmetic industries [82]. It is produced by conversion of isocitrate by isocitrate dehydrogenase and further converted into succinyl CoA by α-ketoglutarate dehydrogenase (α-KD) with consumption of NAD^+^ and CoA. *Y. lipolytica* is unable to synthetize thiamine pyrophosphate, the co-factor of α-ketoglutarate dehydrogenase. Therefore, a culture medium with excess of carbon but lacking thiamine will favor the accumulation of α-KG [50]. The first publication on α-KG production in *Y. lipolytica* (formerly *Candida lipolytica*) dated back to 1969. It reported the production by strain AJ 5004 of α-KG with a titer of 47.2 g/L and a yield from n-paraffins of 0.59 g/g [83]. A C/N ratio ranging from 40:1 to 400:1 in the culture medium is one of the key conditions for α-KG production at high level. Beside n-paraffins, other carbon sources were also used for α-KG production with *Y. lipolytica*, namely glycerol (strain H355, titer 138 g/L, [83]); rapeseed oil (strain VKMY-2412, titer 103 g/L, [84]), cellulose (strain ATCC MYA-2613, 5.5 g/L, [85]), ethanol (strain VKMY-2412, titer 172 g/L, [86]). The DO control is also an important parameter to consider. High DO value (OTR ranging between 0.26–0.56 mmol O_2_/L.min) favor α-KG production [84,86]. Improvement of α-KG production has been also achieved by genetic engineering of producer strains. Overexpression of the *FUM1* gene encoding fumarase (FUM) and *PYC1* encoding pyruvate carboxylase (PYC) in the *Y. lipolytica* strain H355 allowed α-KG titer of 138 g/L within 94 h with a yield of 0.94 g/g glycerol [83]. Overexpression of acetyl-CoA synthase from *S. cerevisiae* (gene *ACS1*) and ATP-citrate lyase from *Mus musculus* (gene *ACL1*) in the *Y. lipolytica* strain WSH-Z06 yielded to a α-KG titer of 56.5 g/L in a 3 L bioreactor within 144 h. This strategy allowed to reduce the intracellular accumulation of pyruvate by increasing the conversion rate of acetyl-CoA [87]. Another strategy with a similar goal was overexpression of a gene encoding pyruvate carboxylase, namely ScPYC1 from *S. cerevisiae* and RoPYC2 from *Rhizopus oryzae* in *Y. lipolytica* WSH-Z06. This resulted in an increased α-KG titer (24.5% and 35.3%, respectively) while pyruvate was reduced by 51.9% and 69.8%, respectively. In a 3 L bioreactor, strain WSH-Z06 with RoPYC2 overexpression produced 62.5 g/L α-KG from 100 g/L glycerol within 144 h [88]. Overexpression of isocitrate dehydrogenase (gene *IDP1*) contributed to a 6.8% increased α-KG titer (167.6 g/L) as compared to the parental strain H355. In strain coexpressing *IDP1* and *PYC1*, the production titer and yield from raw glycerol were 186 g/L and 0.36 g/g [89]. Overexpression of carboxylate transporter (YlJen5p, *YALI0B19470*p) led to a 27.6-fold higher titer (i.e., 46.7 g/L α-KG) and a reduction of pyruvate content of 30.6% (i.e., 12.3 g/L α-KG) [90]. The strain VKMY-2412 was engineered to produce 88.7 g/L α-KG, which was subsequently decarboxylated chemically to succinic acid in the presence of H_2_O_2_ [91]. For additional details on α-KG synthesis in *Y. lipolytica*, refer to the review of Guo et al. (2015) [90].

### 2.5. Itaconic Acid

Itaconic acid (IA) has been classified by the US Department of Energy as one of the 12 value-added bio-based platform chemicals [92]. IA is a petroleum-replacement monomer in plastics and rubbers. In addition, it has applications in detergents, cleaners, and it is considered as a promising alternative to polyacrylic acid. In 2020, the market is expected to exceed 216 million USD. IA is synthesized through the decarboxylation of cis-aconitate, a Krebs cycle intermediate, by cis-aconitic acid decarboxylase (CAD) (Figure 1). Two strategies have been developed for IA production in *Y. lipolytica*. The first one starts from accumulated CA in the cytoplasm, and it is based on the expression of the *Aspergillus terreus* CAD and truncated form of aconitase (i.e., removal of the mitochondrial localization signal in order to have a cytoplasmic enzyme) [92]. With the resulting strain, IA titer of 4.6 g/L was obtained in a 1.5 L bioreactor in 168 h. This corresponded to a yield of 0.058 g/g glucose, and a productivity of 0.045 g/(L·h). The second strategy is based on the direct conversion of cis-aconitate from the Krebs cycle. The latter is first exported in the cytoplasm by mitochondrial tricarboxylic transporter (MTT) before being converted into itaconic acid by recombinant CAD from *A. terreus* [93]. With a recombinant *Y. lipolytica* strain overexpressing CAD and MTT encoding genes, IA titer in a 5 L bioreactor was 22.03 g/L in glucose fed-batch culture with pH not regulated. In those condition, the yield from glucose was 0.064 g/g and productivity of 0.056 g/(L·h).

### 2.6. Acetic Acid

Acetic acid (AA) is an important platform chemical that has a traditional application in the food industry as a preservative. AA is the starting material for the synthesis of vinyl acetate which is used in paints and adhesives, of acetic anhydride used to manufacture textile fibers and cellulose plastics [94]. It is a corrosive carboxylic acid with sour taste and pungent smell. Its chemical synthesis starts from petroleum derived compounds such as butane, methanol, ethylene and acetaldehyde. Its biotechnological production, which accounts for 10% of the worldwide production, mainly relies on lactic acid bacteria. In *Y. lipolytica*, its production as acetate, is catalyzed by acetyl CoA carboxylase, using acetyl CoA as a substrate (Figure 1). The global market of AA is forecasted to reach 16 million tons in 2020 [94].

Some wild-type strains of *Y. lipolytica* have been reported to produce AA under nitrogen limited conditions. This was notably the case for strains AC1-YC-5028 and ACA-YC 5029 grown in a glucose medium (60 g/L) under nitrogen limited conditions (C/N ratio of 200). After 150–200 h of cultivation, AA titer and yield were as maximal as 24 g/L and 0.48 g/g, respectively. At a lower glucose concentration (30 g/L; C/N ratio of 250), such an AA accumulation, was not observed [95]. In those conditions, mainly CA (90–92%, up to 18 g/L) and iCA (8–10%) accumulated in the culture medium. AA was also produced, however in a lower extend (1–2 g/L), by growing strain ACA-DC-50109 in a mixture of OMW (30%) and industrial glucose (65 g/L) [31]. With the same strain, AA was produced, together with other organic acids (iCA, α-KG, malic acid) at 15% *w*/*w* of the total CA amount produced from glucose (42.9 g/L) [29]. AA was also produced as a side (unwanted) metabolite in processes of SA synthesis. It was produced with a titer of 1.36 g/L from rapeseed oil using the *Y. lipolytica* strain VKM Y-2412 [79]. In another process using a recombinant SDH negative *Y. lipolytica* strain for SA synthesis, an acetic acid overflow was observed. It was caused by the transfer of CoA from acetyl-CoA to succinate in the mitochondria rather that pyruvate decarboxylation redaction [77]. In such strain, AA accumulated around 7 g/L after 48 h of culture on glycerol (20 g/L).

## 3. Sugar Alcohol

Sugar alcohols (polyols) are formed by the reduction of their aldo or keto group to a hydroxyl group [96]. *Y. lipolytica* produces polyols, mainly erythritol and mannitol. In the environment, *Y. lipolytica* produces erythritol in response to osmotic stress [1,97]. Metabolites derived from erythritol such as erythrulose and threitol will be also discussed in Section 3.1 to Section 3.3. In *Y. lipolytica*, the mannitol cycle is suggested to supply NADPH for the synthesis of fatty acids [98]. Therefore, mannitol synthesis is related to fatty acid metabolism.

### 3.1. Erythritol

**Figure 2:**
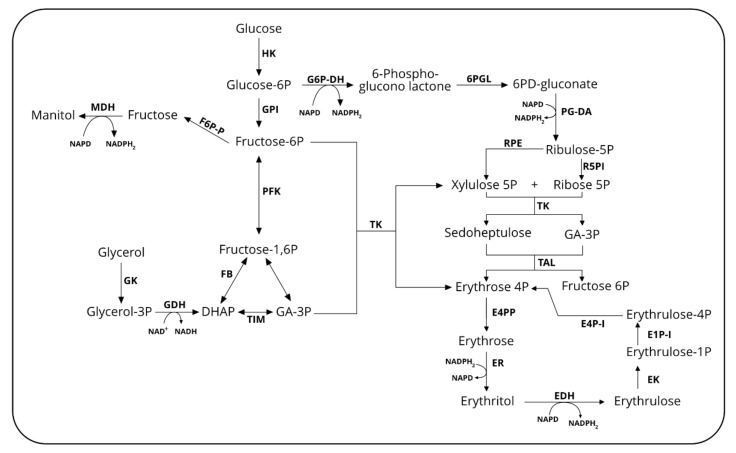
Overview of the principal metabolic pathways for polyols and derivatives synthesis in *Y. lipolytica*. DHAP: dihydroxyacetone-P; *E4PP: Erythrose 4P phosphatase;* ER: erythrose reductase; EDH: erythritol dehydrogenase; EK: erythritol kinase; E1PI: erythrulose-1P isomerase; E4PI: erythrulose-4P isomerase; FBA: fructose-bisphosphate aldolase; *F6P-P: Fructose-6P phosphatse;* GK: glycerol kinase; G3PDH: glycerol-3P dehydrogenase; GPI: glucose-6P isomerase; G6PDH: glucose-6P dehydrogenase; HK: hexokinase; MDH: mannitol dehydrogenase; PFK: phosphofructo kinase; PGDH: phopshogluconate dehydrogenase; RPE: Ribulose-P3 epimerase; R5PI: ribose-5P isomerase; TK: transketolase; TIM: triose isomerase; TAL: transaldolase; 6PGL: 6-phosphonogluconolactonase.

Erythritol (EOL) is a four-carbon sugar alcohol produced as an osmoprotectant by several microorganisms, including *Y. lipolytica*. Like other polyols such as sorbitol, xylitol, mannitol and maltitol, it has sweetening properties with texture and taste of table sugar. EOL is not metabolized by the human body, and thus it is non-caloric and does not modify the glycemia index. EOL is also not cariogenic since it is not metabolized by bacteria responsible for dental caries [97]. In *Y. lipolytica* and other osmotolerant yeasts, EOL synthesis starts from erythrose-4P, an intermediate of the pentose phosphate pathway (PPP) (Figure 2). The latter is dephosphorylated by a still non-characterized erythrose-4P phosphatase (E4PP) before being reduced by an erythrose reductase (ER) into erythritol. In *Y. lipolytica*, three genes encoding erythrose reductase have been characterized so far (namely, *YALI0F18590g, YALI0D07634g* and *YALI0C13508g*) [99,100]. We also recently elucidated the full EOL catabolic pathway [101]. It is first converted into erythrulose by an erythritol dehydrogenase (EYD1, *YALI0F01650g*) and subsequently phosphorylated into L-erythrulose-1P by an erythrulose kinase (EYK1, *YALI0F01606g*). The next step consists in the isomerization into D-erythrulose-4P by an erythrulose-1P isomerase (EYL1, *YALI0F01584g*). In the final step, erythrose-4P, is generated by the activity of an erythrulose-4P isomerase (EYL2, *YALI0F01628g*). Gene *YALIF01562g* (named *EUF1*, as *E*rythritol *U*tilization *F*actor 1) has been characterized recently as essential for efficient EOL assimilation [102]. Its disruption almost impairs the growth of *Y. lipolytica* on EOL used as a sole carbon source. All the genes involved in EOL catabolism were found located on chromosome F in the so called erythritol utilization cluster [103].

EOL synthesis and production strategies in bioreactors have been recently reviewed by our groups and others [97,104,105]. Therefore, only the main achievements that are not covered in these reviews will be presented in this section. EOL productivity has been increased by overexpressing genes encoding erythrose reductase (ER) that convert erythrose into erythritol. By overexpressing the ER gene *YALI0F18590g* in the *Y. lipolytica* strain AMM, Janek et al. (2017) obtained a 20% increase in EOL titer as compared to the parental strain (44.4 g/L) [99]. With that recombinant strain, a productivity of 0.77 g/(L·h) was achieved with a yield from glycerol of 0.44 g/g. After disruption of the gene *YALI0F18590g*, the authors observed a decrease in erythritol titer, suggesting that other isoenzymes were able to convert erythrose into EOL. Recently, we reported on the characterization of genes *YALI0D07634g* and *YALI0C13508g* encoding two additional ERs [100]. With strain CGMCC7326 overexpressing the three known ERs, an EOL titer of 178 g/L was obtained from the medium with an initial glucose of 300 g/L within 84 h with a productivity of 2.1 g/(L·h) and a yield of 0.59 g/g. ERs have been characterized as NADPH-dependent enzymes, therefore their activity could impair co-factor metabolism leading to a lack of available co-factors for enzyme activity. Therefore, the co-factor metabolism was engineered by overexpression of genes encoding glucose-6P dehydrogenase (*ZWF1, YALI0E22694*) and 6-phosphogluconate dehydrogenase (*GND1, YALI0B15598g*) as the corresponding enzyme are known to generate NADPH from NADP^+^ (Figure 2). With the resulting strain HCY108, EOL titer was increased up to 190 g/L within 80 h with a productivity and a yield of 2.4 g/(L·h) and 0.63 g/g glucose, respectively. Other strategies of metabolic engineering consisted of overexpressing key genes of the PPP, namely *TKL1* (*YALI0E06479g*) encoding transketolase, gene *TAL1* (*YALI0F15587g*) encoding transaldolase (Figure 2). In *Y. lipolytica* strain Po1d, overexpression of *TKL1* led to a 19% and 17% increased EOL titer and productivity (43 g/L, 0.04 g/(g_DCW_·h), respectively) [106]. A similar strategy in the strain MK1, led to 51% and 47% increases (51 g/L, 0.53 g/(L·h), respectively) [107]. In the same strain, overexpression of *TAL1* led to an increase in EOL titer and productivity of 45% and 46%, respectively (46.7 g/L and 0.5 g/(L·h)) [107]. These authors also overexpress genes *GND1* and *ZWF1* in strain MK1 and obtained an EOL titer increased by 38% (40.2 g/L) and 41% (42.5 g/L). Non-directed mutagenesis such as NTG or UV mutagenesis has been also used in the past [97]. Beside this, atmospheric and room temperature plasma mutagenesis was also used to generate mutants of *Y. lipolytica* with increased EOL productivity. With such a mutant strain (M53), glycerol fed-batch cultivation in a 5 L bioreactor led to an EOL titer of 169.3 g/L within 168 h with a yield of 0.65 g/g [108].

Fermented okara is a soybean residue, which was used as a feedstock for the production of EOL by mutant strain M53. This allowed an EOL titer of 14.7 g/L with a yield of 0.49 g/g in a 5 L bioreactor within 144 h [108]. By overexpressing *SUC2* genes from *Saccharomyces cerevisiae* encoding invertase and native *GUT1* gene (*YALI0F00384g*), encoding glycerol kinase (GK), EOL was produced from raw industrial molasses (60 g/L) and crude glycerol (100 g/L) and reached a titer of 100 g/L with a productivity and yield of 1.1 g/(L·h) and a yield of 0.67 g/g [109].

By overexpressing genes *GUT1* and *GUT2* (*YALI0B02948g*) encoding glycerol-3-P dehydrogenase (G3P-DH), an EOL titer of 78 g/L was obtained in a 5 L bioreactor within 72 h starting from an initial glycerol of 100 g/L [41]. Beside this, overexpression of genes *GUT1* and *TKL1* in a strain disrupted for gene *EYK1* allowed an erythritol titer of 80 g/L within 192 h with productivity of 1.03 g/(L·h) and a yield of 0.53 g/g glycerol [106]. Polyethylene glycol (PEG) has been used also to mimic osmotic pressure in replacement of NaCl usually added in an EOL production medium. Although the positive effect of NaCl on osmotic pressure, it is known as a corrosive agent of stainless-steel bioreactor vessels and pipes. For the *Y. lipolytica* strain W29, EOL titer was increased by 40% (42 g/L and 0.39 g/g) in the presence of PEG6000 as compared to the medium with NaCl [110]. Trials of EOL production by solid state fermentation (SSF) have been performed with *Y. lipolytica* mutant strain M53 disrupted for gene *SNF1*, a regulator of lipid accumulation. Using peanut press cake mixed with 40% sesame meal and 10% waste cooking oil, an EOL production of 185.4 mg/g was obtained after 96 h [111]. Very recently, EOL was produced at a 500-L scale in raw glycerol fed-batch mode with a titer of 180 g/L after 144 h of cultivation and a resultant yield of 0.53 [112]. Using the same strain, *Y. lipolytica* Wratislavia K1 in a 20 L bioreactor operated in fed-batch titer of 165 g/L was obtained within 146 h with a yield of 0.52 g/g. This study clearly demonstrates that the EOL production process from raw materials is fully scalable.

### 3.2. Erythrulose

As stated above, erythrulose (EOSE) is a tetrose derived from EOL by the action of erythritol dehydrogenase. It has different applications as precursor in chemistry and in cosmetic as a sunless tanning agent [113]. EOSE is involved in the synthesis of the anticancer drug Bengamide E, the antifungal compound Tanikolide, substituted β-lactams, the cytokine modulator Cytoxazone, the cholesterol-lowering drugs Crestor and Zetia, as well as the antiepileptic and hypotensive drug γ-amino-8-hydroxy butyric acid (GABOB) [113]. As a skin tanning agent, EOSE reacts with skin keratin through Maillard reactions creating a tawny effect [113]. In *Y. lipolytica,* disruption of the *EYK1* (*YALI0F01606g*) gene encoding erythrulose kinase impairs the conversion of EOSE into L-erythrulose-1P. Therefore, a strain *Δeyk1* is able to convert EOL into EOSE, and the conversion rate was increased by overexpression of *EYD1* encoding erythritol dehydrogenase. With such a strategy, an efficient fed-batch bioreactor process for the bioconversion of EOL was developed. It permitted a conversion rate of 0.116 g/g_DCW_·h and with a conversion yield of 0.64 g/g [113]. In this process, the ratio of glycerol use as an energy source and EOL seems to be a key parameter of the process.

### 3.3. Other Polyols

Threitol (TOL) is a diastereosisomer of erythritol that is produced by the fungus *Armillaria mellea* and the Alaskan beetle *Upis ceramboides*, where it serves as an antifreeze agent. It has been applied as a precursor for the synthesis of treosulfan used for patients with ovarian cancer and the anticancer drug threitol ceramide. It is also a constitutive element of oxygen sensitive pigment incorporated in smart plastic film used for food packaging [114]. We have recently found that gene encoding xylitol dehydrognase (*Ss-XDH)* from *Schefferomyces stipites* CBS6054 was able to oxidize EOL into EOSE irreversibly, and then reduce EOSE into threitol [114]. By overexpression of the *Ss-XDH* gene in the *Y. lipolytica* strain CGMCC7326, known for its high ability to produce EOL from glucose, a TOL titer of 112 g/L with a yield of 0.37 g/g glucose within 96 h.

Mannitol (MAN) is a six-carbon sugar alcohol that plays an important role in stress tolerance in microorganisms [115]. A recent study suggested that mannitol could accumulate constitutively in cells, which could be involved in scavenging of reactive oxygen species (ROS) generated in some extreme growth condition [116]. Its role in fatty acid metabolism was also reported [98]. Three strains of *Y. lipolytica* (A UV’1, A-15 and Wratislavia K1) were tested for mannitol production from a glycerol-based medium in a bioreactor batch and fed-batch cultures [117]. Besides EOL, a production of mannitol ranging from 2.6 to 14.9 g/L was obtained in a shake-flask culture on crude glycerol. With glycerol fed-batch culture, a strain A UV’1 and A-15 produced mannitol with a titer of 38.1 and 41.4 g/L, respectively, within 150 h. This corresponds to a productivity and yield of 0.29 g/(L·h) and 0.15 g/g; and 0.28 g/(L·h) and 0.17 g/g, respectively [117]. With a mutant strain AIB pAD-UTGut, a derivative of strain AIB that overexpressed *GUT1* gene, a mannitol titer of 11 g/L was obtained in a batch bioreactor on a mixture of crude glycerol and molasses [109]. With the *Y. lipolytica* strain ACA-DC 5029 grown on crude glycerol (70 g/L) blended with OMW, a mannitol titer of 13 g/L in 140 h with a yield of 0.21 g/g glycerol was obtained [118].

Arabitol (ARA) is a five-carbon sugar alcohol with applications as a sweetener in the food industry, as an anticariogenic agent and adipose tissue reducer. It could be used also for the synthesis of chemicals such as xylonic acid, ethylene or propylene [119]. With strain A UV’1 and A-15, ARA titer ranging from 0.3 to 5.6 g/L were obtained from crude glycerol in a shake flask culture [117]. For an engineered strain AIB pAD-UTGut, a derivative of strain AIB that overexpressed *GUT1* gene, an arabitol titer of 7.5 g/L was obtained in a batch bioreactor using a mixture of crude glycerol and molasses [109]. When OMW was blended with 70 g/L glycerol, the *Y. lipolytica* strain ACA-DC 5029 produced ARA with a titer of 4.5 g/L in 264 h with a yield of 0.05 g/g glycerol [118].

## 4. Conclusions and Prospect

*Y. lipolytica* is considered as an emerging cell factory for the production of organic acids and polyols with applications in various economic sector. For commercial production of bulk chemicals using fermentation, the titers above 100–150 g/L are typically required [120]. Table 1 and Table 2 list examples of engineered strains that reached such titers. However, the processes that have been developed so far are still at the laboratory-scale. Hence, the next priority should rely on process industrialization with special emphasis on using raw materials as feedstock in order to reduce the production cost.

## Figures and Tables

**Table 1:** Process parameters of the most promising organic acids produced by *Y. lipolytica*. SSF: solid state fermentation; RBC: repeated batch culture; *is*FBB: *in situ* fibrous bed bioreactor; FB: fed-batch.

Metabolite	Substrate	*Y. lipolytica* Strain	Process	Production Parameters	Reference
Citric acid	Pineapple waste	NCIM3589	SSF	202 g/kg	[30]
	Inulin	AWG7 INU8	RBC	203 g/L, 0.51 g/(L·h), 0.85 g/g	[37]
Iso-citric acid	Ethanol	VKMY-2373	RBC	109 g/L, 1.35 g/(L·h), 0.8 g/g	[54]
Succinic acid	Raw glycerol	PGC01003	*is*FBB	209 g/L, 0.65 g/(L·h), 0.42 g/g	[81]
	Okare	NCYC2904	SSF	34 g/kg	[80]
α-ketoglutarate	Raw glycerol	H355	Batch	186 g/L, 1.75 g/(L·h), 0.36 g/g	[85]
Itaconic acid	Glucose	Po1f	FB	22 g/L, 0.05 g/(L·h), 0.06 g/g	[93]

**Table 2:** Process parameters of the most promising sugar alcohol and derivatives produced by *Y. lipolytica* SF: shake-flask; SSF: solid state fermentation; RBC: repeated batch culture; *is*FBB: *in situ* fibrous bed bioreactor; FB: fed-batch; CG: crude glycerol.

Metabolite	Substrate	*Y. lipolytica* Strain	Process	Production Parameters	Reference
Erythritol	Glucose	CGMCC7326	SF	190 g/L, 2.4 g/(L·h), 0.63 g/g	[100]
	Peanuts cake	M53	SSF	185 g/kg	[111]
Erythrulose	Erythritol	RIY210	FB	0.12 g/(g_DCW_·h), 0.64 g/g	[113]
Threitol	Glucose	CGMCC7326	Batch	112 g/L, 0.37 g/g	[114]
Mannitol	CG/molasses	AIB pAD-UTGut	Batch	13 g/L, 0.21 g/g	[109]
Arabitol	CG/molasses	AIB pAD-UTGut	Batch	5.6 g/L	[1]

## Abbreviations

AA	acetic acid
ARA	arabitol
KD	α-ketoglutarate dehydrogenase
α-KG	α-ketoglutarate
6PGL	6-phosphonogluconolactonase
ACC	acetyl CoA carboxylase
ACH	acetyl CoA hydrolase
ACL	ATP citrate lyase
ACO	aconitase
ACO *	truncated aconitase
ARA	arabitol
CA	citric acid
CAD	cis-aconitate decarboxylase
CIT	citrate synthase
DHAP	dihydroxyacetone-P
DO	dissolved oxygen
E1PI	erythrulose-1P isomerase
E4PI	erythrulose-4P isomerase
E4PP	erythrose 4P phosphatase
EDH	erythritol dehydrogenase
EK	erythritol kinase
EOL	erythritol
EOSE	erythrulose
ER	erythrose reductase
F6P-P	fructose-6P phosphatse
FBA	fructose-bisphosphate aldolase
iCA	isocitric acid
*is*FBB	*in situ* fibrous bed bioreactor
FUM	fumarase
G3PDH	glycerol-3P dehydrogenase
G6PDH	glucose-6P dehydrogenase
GK	glycerol kinase
GPI	glucose-6P isomerase
HK	hexokinase
IA	itaconic acid
iCA	iso-citric acid
ICL	isocitrate lyase
IDH	isocitrate dehydrogenase
MAN	mannitol
MDH	mannitol dehydrogenase
MLS	malate synthase
NTG	N-methyl-N’-nitro-N-nitrosoguanidine
OMW	olive-mill wastewater
PCK	phosphoenolpyruvate carboxykinase
PEP	phosphoenol pyruvate
PFK	phosphofructo kinase
PGDH	hopshogluconate dehydrogenase
PYC	pyruvate carboxylase
R5PI	ribose-5P isomerase
RPE	ribulose-P3 epimerase
SA	succinic acid
SCS	succinyl CoA synthetase
SDH	succinate dehydrogenase
SSF	solid-state fermentation
TAL	transaldolase
TIM	triose isomerase
TK	transketolase
TOL	threitol
UV	ultra-violet
